# Starting Editorial of “Cellular Damage: Protection and Induction” Addressing Hot Topics in Cellular Damage, Protection of Cells and Therapy Targeting Bad Cells

**DOI:** 10.3390/ijms241813702

**Published:** 2023-09-05

**Authors:** Songwen Tan, Wenhu Zhou

**Affiliations:** Xiangya School of Pharmaceutical Sciences, Central South University, Changsha 410013, China

## 1. Introduction

The cell, the fundamental unit of life, is constantly subjected to a myriad of molecular biophysical disturbances [1]. These disturbances can arise from various sources, including the chemical and biophysical techniques used in cryopreservation [2,3]. A deep understanding of cellular damage and its underlying mechanisms is crucial [4]. Equally important is the recognition of the dual nature of our interventions; while we aim to protect cells from harm, there are instances where we intentionally induce damage, especially in clinical settings like cancer treatments [5,6,7].

In this Special Issue titled “Cellular Damage: Protection and Induction”, we aim to shed light on the complex nature of biophysical damage that cells encounter. Our focus spans from the protective strategies employed in regenerative medicine and pharmacology to the intricacies of cryopreservation. In contrast, we also delve into the intentional harms associated with treatments like radiation and chemotherapy. We cordially invite scholars to contribute to this Special Issue with their groundbreaking research and insightful reviews. The topics of interest encompass the intricate molecular pathways leading to biophysical cellular disruption and the evolving strategies that either enhance cellular defenses or deliberately target them. Table 1 shows the suggested topics of interest for the “Cellular Damage: Protection and Induction” Special Issue.

## 2. Cellular Damage

Cellular damage involves harm inflicted on cells through external factors or internal dysfunctions, taking on various forms [8]. This damage can be caused by a myriad of factors, including environmental [9], chemical [10], and biophysical triggers [11].

Chemical and Biophysical Approaches to Cryopreservation: Cryopreservation is a technique used to preserve biological samples, including cells, at extremely low temperatures. The process can introduce chemical and biophysical damage to cells, which can affect their viability upon thawing. Understanding these mechanisms is crucial for improving cryopreservation outcomes [12,13].

Molecular Mechanisms of Biophysical Damage: At the molecular level, biophysical damage can result from various factors, including radiation, mechanical strain, and temperature changes. These damages can lead to protein misfolding, DNA breaks, and cellular dysfunction [14,15].

Environmental and Internal Triggers of Biophysical Strain: Cells are constantly exposed to various environmental factors, such as toxins, pathogens, and physical forces. Internally, metabolic imbalances and genetic mutations can also introduce strain, leading to cellular damage [16]. Innovations in understanding cellular damage are hot topics in foundational study.

## 3. Protection of Cells

Protecting cells from damage is crucial for maintaining tissue and organ function. Advances in understanding cellular damage have paved the way for innovative strategies to shield cells from harm [17].

Regenerative medicine technology: Regenerative medicine focuses on repairing or replacing damaged tissues and organs. Techniques in this field often involve the use of stem cells, growth factors, and biomaterials to promote healing and regeneration [18,19].

Pharmacological Interventions for Cellular Protection: Various drugs and compounds have been developed to enhance cellular resilience and protect cells from damage. These interventions can target specific pathways involved in cellular stress responses.

Cellular Resilience and Recovery Pathways: Cells have intrinsic mechanisms to recover from damage and restore their function. Understanding these pathways can provide insights into developing strategies for cellular protection [20].

## 4. Therapy Targeting Bad Cells

Targeting and eliminating damaged or dysfunctional cells is a promising therapeutic approach for various diseases, including cancer.

Radiation-Induced Cellular Injuries: Radiation therapy is commonly used to treat cancer. However, it can also introduce cellular injuries. Strategies to target and repair these injuries can enhance the therapeutic outcomes [21].

Chemotherapy Strategies: Chemotherapy drugs target rapidly dividing cells, including cancer cells. Understanding the mechanisms of these drugs can lead to the development of more effective and less toxic treatments [22].

Targeted Therapies for Specific Cell Types: Advances in molecular biology have led to the development of targeted therapies that can specifically attack certain cell types or pathways, minimizing damage to healthy cells [23].

Intentional Cellular Disruptions in Therapeutic Settings: In some therapeutic settings, intentional disruption of certain cell populations can be beneficial. For instance, eliminating senescent cells can promote tissue regeneration and reduce inflammation [24].

## 5. Future Prospects

The understanding of cellular damage, protection, and targeted therapies has grown exponentially in recent years. As we continue to unravel the intricate mechanisms underlying these processes, we can anticipate the development of more effective and precise interventions to treat a wide range of diseases. The convergence of biotechnology, pharmacology, and molecular biology will undoubtedly pave the way for groundbreaking therapies that can extend the human healthspan and improve the quality of life.

## Figures and Tables

**Table 1:** Topics of Interest for the “Cellular Damage: Protection and Induction” Special Issue.

Cellular Damage	Protection of Cells	Therapy Targeting Bad Cells
Chemical and biophysical approaches to cryopreservation	Regenerative medicine techniques	Radiation-induced cellular injuries
Molecular mechanisms of biophysical damage	Pharmacological interventions for cellular protection	Chemotherapy strategies
Environmental and internal triggers of biophysical strain	Cellular resilience and recovery pathways	Targeted therapies for specific cell types
Innovations in understanding cellular damage	Advanced strategies for cellular protection and repair	Intentional cellular disruptions in therapeutic settings
